# Reverse engineering module networks by PSO-RNN hybrid modeling

**DOI:** 10.1186/1471-2164-10-S1-S15

**Published:** 2009-07-07

**Authors:** Yuji Zhang, Jianhua Xuan, Benildo G de los Reyes, Robert Clarke, Habtom W Ressom

**Affiliations:** 1 Lombardi Comprehensive Cancer Center, Georgetown University, 4000 Reservoir Rd, Washington, DC, USA; 2 Department of Electrical and Computer Engineering, Virginia Polytechnic Institute and State University, 4300 Wilson Blvd., Arlington, VA, USA; 3 School of Biology and Ecology, University of Maine, Orono, ME 04469, USA

## Abstract

**Background:**

Inferring a gene regulatory network (GRN) from high throughput biological data is often an under-determined problem and is a challenging task due to the following reasons: (1) thousands of genes are involved in one living cell; (2) complex dynamic and nonlinear relationships exist among genes; (3) a substantial amount of noise is involved in the data, and (4) the typical small sample size is very small compared to the number of genes. We hypothesize we can enhance our understanding of gene interactions in important biological processes (differentiation, cell cycle, and development, etc) and improve the inference accuracy of a GRN by (1) incorporating prior biological knowledge into the inference scheme, (2) integrating multiple biological data sources, and (3) decomposing the inference problem into smaller network modules.

**Results:**

This study presents a novel GRN inference method by integrating gene expression data and gene functional category information. The inference is based on module network model that consists of two parts: the module selection part and the network inference part. The former determines the optimal modules through fuzzy c-mean (FCM) clustering and by incorporating gene functional category information, while the latter uses a hybrid of particle swarm optimization and recurrent neural network (PSO-RNN) methods to infer the underlying network between modules. Our method is tested on real data from two studies: the development of rat central nervous system (CNS) and the yeast cell cycle process. The results are evaluated by comparing them to previously published results and gene ontology annotation information.

**Conclusion:**

The reverse engineering of GRNs in time course gene expression data is a major obstacle in system biology due to the limited number of time points. Our experiments demonstrate that the proposed method can address this challenge by: (1) preprocessing gene expression data (e.g. normalization and missing value imputation) to reduce the data noise; (2) clustering genes based on gene expression data and gene functional category information to identify biologically meaningful modules, thereby reducing the dimensionality of the data; (3) modeling GRNs with the PSO-RNN method between the modules to capture their nonlinear and dynamic relationships. The method is shown to lead to biologically meaningful modules and networks among the modules.

## Background

In recent years, high throughput biotechnologies have made large-scale gene expression surveys a reality. Gene expression data provide an opportunity to directly review the activities of thousands of genes simultaneously. However, computational methods that can handle the complexity (noisy, substantial amount of variables, high dimensionality, etc.) of these biological data are often unavailable [1]. Powerful computational methods and data mining tools are needed for biologically meaningful inferences from gene expression data.

Cluster analysis has been used to separate genes into groups based on their expression profiles [2], in which similar expression profiles will be more likely in the same group. Although cluster analysis gives insight into the groups of genes that may share similar functions, the inference of the relationships among these groups is beyond what cluster analysis can do.

A variety of continuous or discrete, static or dynamic, quantitative or qualitative models have been proposed for inference of biological networks. These include biochemically driven methods [3], linear models [4,5], Boolean networks [6], fuzzy logic [7,8], Bayesian networks [9], and recurrent neural networks [10-12]. Biochemically inspired models are developed on the basis of the reaction kinetics between different components of a network. However, most of the biochemically relevant reactions under participation of proteins do not follow linear reaction kinetics, and the full network of regulatory reactions is very complex and hard to unravel in a single step. Linear models attempt to solve a weight matrix that represents a series of linear combinations of the expression level of each gene as a function of other genes, which is often underdetermined since gene expression data usually have far fewer dimensions than the number of genes. In a Boolean network, the interactions between genes are modeled as Boolean function. Boolean networks assume that genes are either "on" or "off" and attempt to solve the state transitions for the system. The validity of the assumptions that genes are only in one of these two states has been questioned by a number of researchers, particularly among those in the biological community. In [7], an approach is proposed based on fuzzy rules of a known activator/repressor model of gene interaction. This algorithm transforms expression values into qualitative descriptors that can be evaluated by using a set of heuristic rules and searches for regulatory triplets consisting of activator, repressor, and target gene. This approach, though logical, is a brute force technique for finding gene relationships. It involves significant computation time, which restricts its practical usefulness. In [8], we propose the use of clustering as an interface to a fuzzy logic-based method to improve the computational efficiency. In a Bayesian network model, each gene is considered as a random variable and the edges between a pair of genes represent the conditional dependencies entailed in the network structure. Bayesian statistics are applied to find certain network structure and the corresponding model parameters that maximize the posterior probability of the structure given the data. Unfortunately, this learning task is NP-hard, and it also has the underdetermined problem. The recurrent neural network (RNN) model has received considerable attention because it can capture the nonlinear and dynamic aspects of gene regulatory interactions. Several algorithms have been applied for RNN training in network inference tasks, such as fuzzy-logic [11] and genetic algorithm [12]. In [10,13], we applied particle swarm optimization (PSO) method to train the RNN for network inference, yielding promising results.

As variant sources of biological data are becoming available now, it is very necessary and helpful to infer gene regulatory network (GRN) not only from one single data source, but from data fusion of multiple complementary data sources. A few previous studies combined time course gene expression data with other data sources, such as genomic location data [14] and sequence motif [15]. Prior knowledge of GRN helps understand gene interactions in important biological processes such as differentiation, cell cycle, and development. Due to the specific properties of gene expression data, the task of inferring GRNs involves several challenges including: (1) living cells contain thousands of genes (high dimensionality); (2) each gene interacts with one or more other genes directly or indirectly with complex dynamic and nonlinear relationships, (3) current technologies generate data that involve a substantial amount of noise, and (4) due to the cost of large-scale gene expression profiling experiments, the sample size is extremely low compared with the number of genes. In this study, we address these challenges by: (1) preprocessing gene expression data (e.g. normalization and missing value imputation) to reduce the data noise; (2) clustering genes with gene expression data and gene functional category information to find the optimal modules with biological significance and reduce the problem dimensionality; (3) modeling GRNs with the particle swarm optimization – recurrent neural network (PSO-RNN) method between the modules to capture their nonlinear and dynamic relationships.

Our previous studies [10,13] demonstrate that we can benefit by incorporating known gene functional category information in terms of improving the inferential power of our framework. Moreover, instead of using fully connected RNN model, we propose a network pruning method to select the statistically significant weights for the final GRN structure using PSO. The hybrid PSO-RNN algorithm is applied to infer networks of interactions from two real-world gene expression data. The inferred GRNs are confirmed with previous studies.

## Results and discussion

In this section, we demonstrate the inference ability of our proposed method via two experimental studies: the rat central nervous system (CNS) and yeast cell cycle process. Both data were preprocessed in the original studies [16,17]. To proceed with the module network inference process, we first imputed the missing values in the data by using the Bayesian principal component analysis (BPCA) method [18]. Following that, we standardized the data between zero and one.

### Rat CNS data

This case study is based on the data published in [16], consisting of gene expression levels for 112 genes during the development of the CNS of rats. Each gene was measured at nine different points in time (of which the last, measured for the adult animal, was not used here). The first measurement was made 10 days before birth, and the intervals between measurements were 2 or 3 days in the period before birth and 7 days after birth. The gene functional category information can also be found in [16].

**Figure 1:**
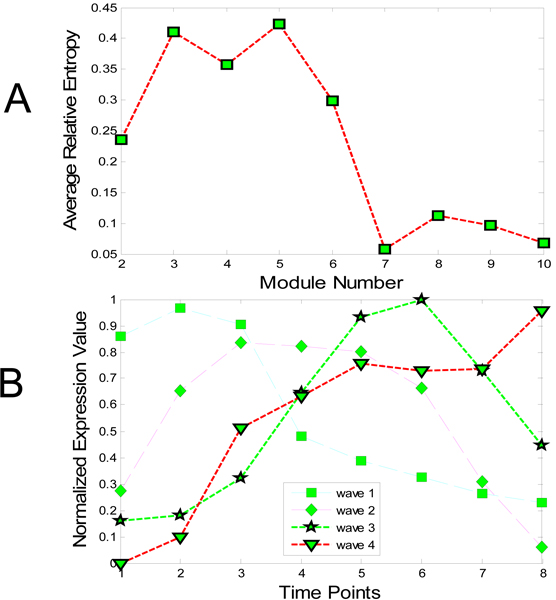
**Module selection of rat CNS data**. The module selection of rat CNS data is shown in these figures: A. Estimate of the optimal number of modules: the optimal number of FCM clustering is five, which agrees with the result presented in [16]. B. Four modules (waves) based on the optimal cluster number in A: the expression levels of the first four clusters are shown (the fifth cluster consists of diverse genes, not used for further analysis).

The module selection result and corresponding modules are shown in Figure 1. As illustrated In Figure 1A, the optimal number of fuzzy c-means (FCM) clustering is five, which confirms previous cluster result reported in [16]. Figure 1B shows the expression levels of the four clusters (the fifth cluster consists of diverse genes, not used for further analysis). We also compared the genes in each cluster with the cluster results in [16]. Most genes are the same in each cluster, although the clustering methods are different in the two studies. We considered four modules that correspond to the first four clusters: Module 1 consists of genes active during initial proliferation, Module 2 contains genes associated with neurogenesis, Module 3 in made up of most genes for neuro transmitter signaling, and Module 4 contains genes active during the final maturation of the tissue. This shows that our module selection method has the ability to identify the optimal number of modules by incorporating gene function category information.

**Figure 2:**
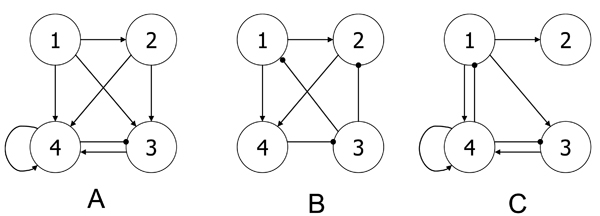
**Comparison of results from three studies**. A. Our method; B. Deng *et al. *(2005) [19]; C. Wahde *et al. *(2001) [20]. Line with arrow: Up regulation; line with dot: down regulation.

**Figure 3:**
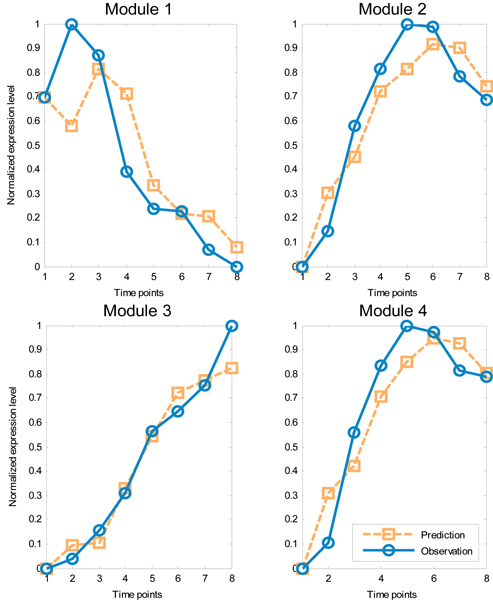
**The time-course of observed expression and prediction for modules of CNS data**.

The reverse engineering algorithm is applied to the four modules for network inference. The final reconstructed network was built by choosing significant parameters as described in the Methods section. Our results were compared to those obtained by Deng *et al. *[19] (Figure 2B) and Wahde *et al. *[20] (Figure 2C). Apparently, the three results agree on certain regulation pathways. Module 1 shows up regulation to Module 2, 3 and 4 in our method, which can be verified in either (b) or (c). The regulation associated with Module 4 can also be found in (b) and (c). Only one new regulation inferred by our method could not be found in (b) and (c): Module 2 up-regulates Module 3. According to Figure 1B, the expression level of Module 2 is apparently followed by Module 3, which confirms the results. Figure 3 shows the time course of observed expressional levels of four modules and their predictions by the inference method.

### Yeast cell cycle data

The yeast cell cycle data presented in [17] consist of six time series (*cln3*, *clb2*, *alpha*, *cdc15*, *cdc28*, and *elu*) expression measurements of the transcript (mRNA) levels of *S. cerevisiae *genes. 800 genes were identified as cell cycle regulated based on cluster analysis in [17]. Here, we used the *cdc15 *time course data of the 800 genes since it has the largest number of time points (24).

**Figure 4:**
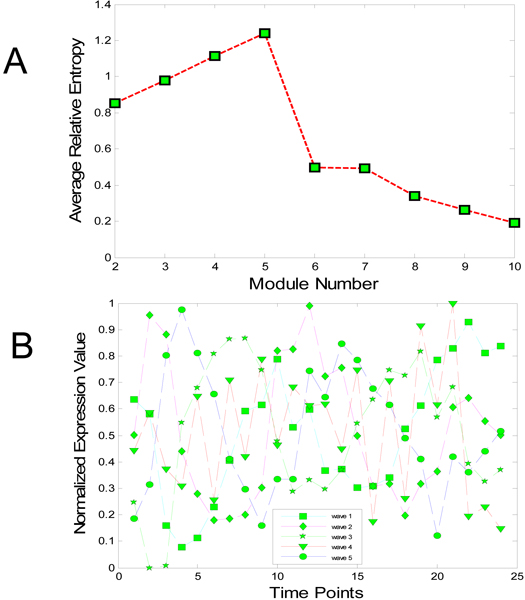
**Module selection of yeast cell cycle data**. A. Estimate of the optimal number of modules; B. Five modules (waves) based on the optimal number in A.

Spellman et al. [17] assigned attributes (called peaks) for genes that represent the time when gene expression levels take the peak during cell cycle. Based on the four phrases in a cell cycle, *G1 *-> *S *-> *G*2 -> *M*, Spellman et al. assigned each gene to one of the five peaks *G*1, *S*, *S/G*2, *G*2/*M*, and *M/G*1. Using this information, we selected the module shown in Figure 4 for the *cdc15 *data set. As shown in Figure 4A, the optimal number of FCM clustering is five, which is based on the number of peak phrases each gene can be assigned to. Table 1 shows the number of genes with different peak time for each cluster. From the highlighted numbers in Table 1, we can characterize the modules: It is clear that Module 1 is responsible for genes with peaks in M/G1 or G1, followed by Module 2, and so on. The expression levels of modules are shown in Figure 4B.

**Table 1:** Mapping of expression clusters to functional gene classes.

	G1	S	S/G2	G2/M	M/G1
wave1	**210**	2	2	2	**25**
wave2	46	**63**	**67**	2	1
wave3	0	1	**38**	**125**	3
wave4	9	1	2	**24**	**58**
wave5	**35**	4	12	26	**42**

This table shows the number of genes with different peak time for each cluster in yeast cell cycle data. From the highlighted numbers in the table, we can characterize the modules: It is clear that Module 1 is responsible for genes with peaks in M/G1 or G1, followed by Module 2, and so on.

**Figure 5:**
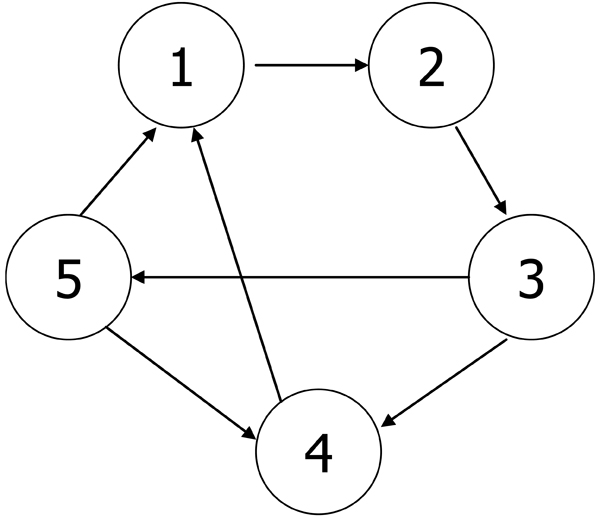
**Inferred yeast module network**. All the regulations identified in yeast module network are positive. Considering such characteristics of the modules and directions of the arcs between modules, the obtained network is believed to code a partially consistent regulatory relationship between modules recalled from the time sequence of the phase in cell cycle. All the relationships among modules indicate that each module has some regulatory impact on its follow-up modules, according to the peaks each module stands for. There is one exception: Module 5 has an up-regulation on Module 4, which shows that some feedback may exist in yeast cycle process.

**Figure 6:**
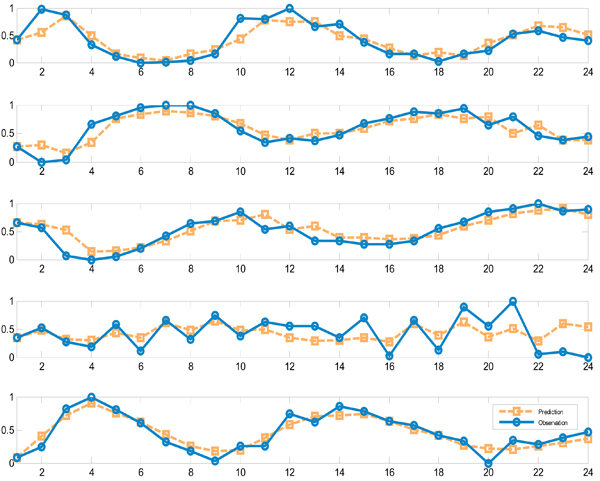
**The time-course of observed expression and prediction for modules of *cdc15 *data**.

The PSO-RNN algorithm is applied to the network inference of the five modules. The final reconstructed network is inferred by choosing significant parameters as described in the Methods section. Unlike the CNS data, we could not compare our results to other publications due to lack of similar studies. Instead we illustrate the results according to their peak attributes. As shown in Figure 5, all the regulations identified here are positive. Considering such characteristics of the modules and directions of the arcs between modules, we observe that the obtained network codes a partially consistent regulatory relationship between modules recalled from the time sequence of the phase in cell cycle. All the relationships among modules indicate that each module has some regulatory impact on its follow-up modules, according to the peaks each module stands for. There is one exception: Module 5 has an up-regulation on Module 4, which shows that some feedback may exist in yeast cycle process. Figure 6 shows the time course of observed expressional level for five modules and their predictions by the inference method.

## Conclusion

Reverse engineering of GRNs from time course gene expression data is a major obstacle in system biology due to the limited number of time points. We demonstrate that our method can address this challenge by decomposing the reverse engineering problem into modules, where two steps are involved: the gene expression data is clustered into modules with biological significances to reduce the problem dimensionality, and the network is built based on the expression profiles of modules. We evaluate the performance of the algorithm using two real data sets: rat CNS data and yeast cell cycle data. The results indicate that biologically meaningful modules are selected and biologically plausible networks between modules are estimated. For example, in CNS data, the inferred network at module level is a combination of the networks verified in the other two studies [19,20]. Our future research will focus on network module inference with more detailed gene category/regulation information. Multiple data sources (e.g. ChIP-on-Chip data [21], motif information, and gene ontology annotation) can be used for this purpose. Also, the data fusion from complementary data sources will not only help solve the underdetermined problem in GRN inference, but also increase the prediction accuracy. Another direction to address the under-determined reverse engineering problem is to decompose the GRN into small subnetworks, called network motifs (NMs) [22].

## Methods

The proposed method includes two parts: module selection and network inference. In the module selection part, we cluster the genes by FCM clustering. The optimal number of clusters is determined by the relative entropy estimate method, which incorporates the gene functional category information; each cluster is considered as a module representing certain co-regulated genes. After the modules are determined, the PSO-RNN inference algorithm is applied. In this algorithm, each module is considered as a neuron in the RNN structure, and any regulation between two modules is a weight in the RNN. To find the best fit network among the modules, a generalized PSO method, including basic PSO and neural network pruning technique, is used to determine RNN structure and its parameters.

### Module selection

Clustering has been a major method to partition the genes into groups of co-expressed genes [23]. However, most of these clustering methods are purely data-driven with no prior biological knowledge. Here we present a new clustering method based on FCM clustering. Instead of using purely data-driven estimate methods, we propose a new estimate method to select the optimal number of clusters by incorporating gene functional category information.

#### FCM clustering

FCM is a method of clustering which allows a data point to belong to two or more clusters. The detailed description of FCM method can be found in [24]. Several methods have been used for estimating the optimal number of clusters, e.g. Xie-Beni statistic [24]and gap statistic [25]. Because all these methods are purely data-driven, it is not suitable to estimate the clustering of gene expression data.

#### Estimating the number of modules

We propose a new computational method to determine the number of biologically meaningful modules. This is accomplished by incorporating gene functional category information into FCM cluster analysis and applying the relative entropy to measure the biological significance of a cluster to serve as a network module. The relative entropy *D*(*p*||*q*) is a measure of the inefficiency of assuming that the distribution is *q *when the true distribution is *p*. For one discrete random variables *x *with two different distributions *p *and *q*, the relative entropy between them is defined as

(1)

where Λ is the sample space of *x*. The goal is to identify the clusters with significant relative entropy.

In a gene expression data set, all genes can be characterized into some categories according to their functions or other properties (e.g. gene peak phase in cell cycle process). For example, according to the gene functional category information, we can get the probability distribution of category for the data set (say *p*). After the FCM clustering, the probability distribution of category for each cluster can also be obtained (say *q*). We want to know how different *p *and *q *are. The more different they are, the more significant the cluster (corresponding to *q*) is. The procedure is defined as follows: let *C *be the total number of clusters obtained from the FCM clustering (*C *= 2, 3,..., 10). For each *C*, we calculate the relative entropy of *p *and *q*_*i *_(*i *= 1, 2,..., C). The average of the relative entropies *ave(D*_*C*_) in one FCM clustering, defined in (2), is considered as the estimate for the number of clusters. The number of clusters with maximum *ave(D*_*C*_), defined in (3), is considered as the optimal module number C*. With the optimal module number C*, we cluster the gene expression data. Each cluster center represents the expression profile for its own module, which is subject to the network inference among modules.

(2)

(3)

### Network inference

In building an RNN to infer a network of interactions, the identification of the correct structure and determination of the free parameters (weights and biases) to mimic measured data is a challenging task given the limited available quantity of data and complex search space. In this paper, we apply PSO and neural network pruning methods to select the optimal architecture of an RNN and update its free parameters.

#### Network model

The genetic regulation model can be represented by a recurrent neural network formulation [5,20]:

(4)

where *x*_*i *_is the gene expression level of the *i*^*th *^gene (1 ≤ *i *≤ *N*), *N *is the number of genes in the model), *φ*(·) is a activation function, *w*_*ij *_represents the effect of *j*^*th *^gene on the *i*^*th *^gene (1 ≤ *i*, *j *≤ *N*), *b*_*i *_denotes the bias for the *i*^*th *^gene, and *τ *is the decay rate parameter. The function *φ *(·) introduces nonlinearity to the model.

When information about the complexity of the underlying system is available, a suitable activation function can be chosen (e.g. linear, logistic, sigmoid, threshold, hyperbolic tangent sigmoid or Gaussian function.) If no prior information is available, our algorithm uses by default the sigmoid function. A negative value of *w*_*ij *_represents the inhibition of the *j*^*th *^gene on the *i*^*th *^gene, whereas a positive value of *w*_*ij *_represents the activation control of the *j*^*th *^gene on the *i*^*th *^gene. If *w*_*ij *_is zero, then it means that the *j*^*th *^gene has no influence on the *i*^*th *^gene. The discrete form of (1) can written as

(5)

**Figure 7:**
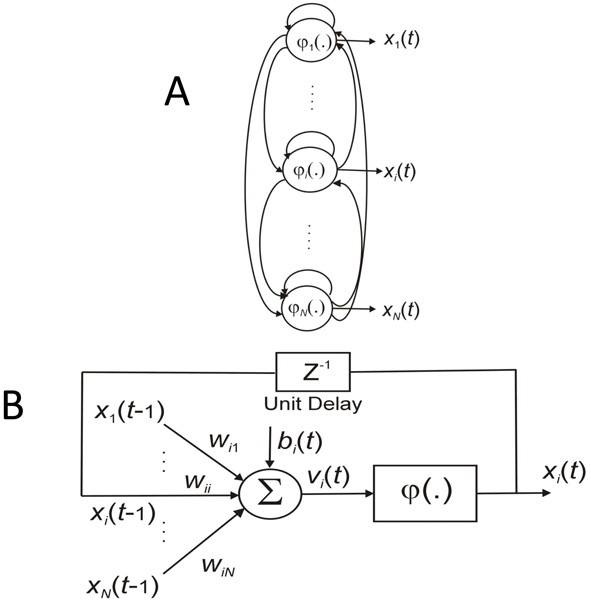
**The description of a GRN by a RNN model**. A: A fully connected RNN model, where the output of each neuron is fed back to its input after a unit delay and is connected to other neurons. It can be used as a simple form mimicking a NM, where a gene cluster or a TF is represented by a neuron. B: Details of a single recurrent neuron.

Figure 7 shows the architecture of a RNN that can simulate the mathematical relationship in Eq. (5). As illustrated in the figure, the output of each neuron is fed back to its input after a unit delay and is connected to other neurons [26]. It can be used as a simple form of GRN module, where each entity (e.g. gene or module) in the network is considered as a neuron. The RNN can model not only the interactions between entities but also entity self-regulation.

Training the RNNs involves determining the optimal weights *w*_*ij *_and bias *b*_*i*_. As a cost function, we use the mean-squared error between the expected output and the network output across time (from the initial time point 0 to the final time point *T*) and across all *N *neurons in the network. The cost function can be written as:

(6)

where *x*_*i*_(*t*) and  are the true and predicted values (expression levels) for the *i*^*th *^neuron (entity) at time *t*. The goal is to determine the structure and weights that minimize this cost function.

#### Training algorithm

There exist many algorithms for RNN training in the literature, e.g., back-propagation through time (BPTT) [27] and genetic algorithm (GA) [12]. BPTT is an extension of the standard back-propagation algorithm, using gradient descent method to find the best solution. However, the use of the gradient descent requires the error function to be differentiable, and also makes the procedure easy to get stuck in local minima. GA, inspired by the natural evolution process, has been applied to optimize the GRN in some applications [12,28].

Here, we use PSO [29] for RNN structure training. It has been shown that PSO requires less computational cost and can achieve faster convergence than conventional back-propagation in training neural networks for approximating a nonlinear function [30]. Compared with GA, PSO is easy to implement and there are few parameters to adjust. Particularly, PSO has memory for the previous best solutions to avoid the possible loss of learned knowledge. All these features make PSO suitable for GRN inference.

In PSO, each particle is represented as a vector  and instantaneous trajectory vector , describing its direction of motion in the search space at iteration *k*. The index *i *refers to the *i*^*th *^particle. The core of the PSO algorithm is the position update rule (7) which governs the movement of each of the *n *particles through the search space.

At any instant, each particle is aware of its individual best position, , as well as the best position of the entire swarm, . The parameters *c*_1 _and *c*_2 _are constants that weight particle movement in the direction of the individual best positions and global best positions, respectively; and *r*_1, *j *_and *r*_2, *j*_, *j *= 1, 2,... *D *are random scalars distributed uniformly between 0 and 1, providing the main stochastic component of the PSO algorithm.

(7)

where



The constriction factor *χ *may also help to ensure convergence of the PSO algorithm, and is set according to the weights *c*_1 _and *c*_2 _as in (8).

(8)

The key strength of the PSO algorithm is the interaction among particles. The second term in (7), , is considered to be a "social influence" term. While this term tends to pull the particle towards the globally best solution, the first term, , allows each particle to think for itself. The net combination is an algorithm with excellent trade-off between total swarm convergence, and each particle's capability for global exploration. Moreover, the relative contribution of the two terms is weighted stochastically.

The algorithm consists of repeated application of the velocity and position update rules presented above. Termination can occur by specification of a minimum error criterion, maximum number of iterations, or alternately when the position change of each particle is sufficiently small as to assume that each particle has converged.

Selection of appropriate values for the free parameters of PSO plays an important role in the algorithm's performance. The parameter setting we used in this study can be found in Table 2, which are the default values in PSOt toolbox [31]. The maximum search space range defines the maximum allowed values of each element in one particle.

**Table 2:** PSO Parameter setting

Parameter	Value
Maximum search space range, |*W*_*max*_|	[-5, 5]
Acceleration constants, *c*_1 _&*c*_2_	2.05, 2.05
Size of swarm	50–150

#### PSO-RNN hybrid algorithm

In this section, we illustrate how PSO optimizes the parameters of RNN and how the structure of RNN is pruned to mimic the response of an unknown network of interactions. Since PSO is a stochastic algorithm, a single solution may not reflect the underlying network. We therefore collect a number of solutions from the PSO-RNN algorithm and use them to determine a single output network that receives the majority vote. Specifically, we applied 100 runs for each network inference. If the absolute value of the average of one parameter in hundred runs is larger than its standard deviation, it is said significant and will be selected for the final network, otherwise it will be set to zero. The following reverse engineering procedure is utilized:

1. Run the reverse engineering algorithm without introducing any particular constraints (except the maximum-allowed values) in the network parameters. Perform hundred runs, and select the networks with mean squared error (MSE) less than certain threshold for further network parameter evaluation.

2. Determine the average and standard deviations of the network parameters using the results from Step 1.

3. Set non-significant parameters (if any) to zero. If there is no non-significant parameter, the procedure is stopped.

4. Return to the reverse engineering algorithm, with non-significant weights set to zero. If the results (measured by the fitness) are as good, or almost good, as for the previous sets of runs, form the network averages, and return to Step 3. If instead the results are worse than in the previous run, discontinue the procedure.

**Figure 8:**
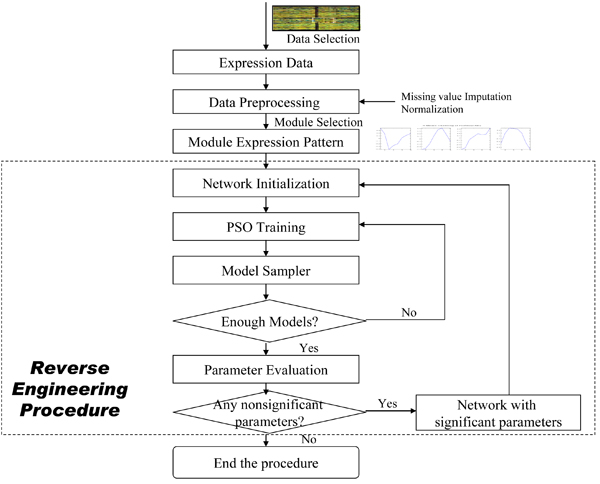
**The flowchart of the proposed approach**. The flowchart of the proposed approach is illustrated here, involving mainly two components: (1) Module selection is performed after data preprocessing (including missing value imputation and normalization) to produce the module expression patterns; (2) the reverse engineering procedure PSO-RNN determines both the structure and corresponding parameters of a RNN which represents the underlying structure of a module network.

Concluding all the above process, the overall algorithm is illustrated in Figure 8, which involves mainly two components: (1) Module selection is performed after data preprocessing (including missing value imputation and normalization) to produce the module expression patterns; (2) the reverse engineering procedure PSO-RNN determines both the structure and corresponding parameters of a RNN which represents the underlying structure of a module network.

## List of abbreviations used

(CNS): Central nervous system; (FCM): fuzzy c-means; (GRN): Gene regulatory network; (MSE): mean square error; (NM): network motif; (PSO): particle swarm optimization; (RNN): recurrent neural network.

## Competing interests

The authors declare that they have no competing interests.

## Authors' contributions

Y. Zhang and H.W. Ressom designed the computational approach, wrote the code, analyzed the experimental results, and drafted the manuscript. All authors read and approved the final manuscript.
